# Mortality Predictors in the Surgical Treatment of Active Infective Endocarditis

**DOI:** 10.21470/1678-9741-2017-0132

**Published:** 2018

**Authors:** Jenny Lourdes Rivas de Oliveira, Magaly Arrais dos Santos, Renato Tambellini Arnoni, Auristela Ramos, Dorival Della Togna, Samira Kaissar Ghorayeb, Roberto Tadeu Magro Kroll, Luiz Carlos Bento de Souza

**Affiliations:** 1 Instituto Dante Pazzanese de Cardiologia, São Paulo, SP, Brazil.

**Keywords:** Endocarditis, Bacterial, Heart Valves, Cardiovascular Surgical Procedures, Heart Valve Prosthesis Implantation, Mortality

## Abstract

**Introduction:**

Active infective endocarditis is associated with high morbidity and mortality. Surgery is indicated in high-risk conditions, and the main determinants of mortality in surgical treatment should be evaluated.

**Objective:**

To identify mortality predictors in the surgical treatment of active infective endocarditis in a long-term follow-up.

**Methods:**

This prospective observational study involved 88 consecutive patients diagnosed with active infective endocarditis, who underwent surgery between January 2005 and December 2015. Fifty-eight (65.9%) patients were male, the mean age was 50.87±16.15 years. A total of 31 (35.2%) patients had a history of rheumatic fever; 48 (54.5%) had had heart surgery with prosthetic valve implantation; 45 (93.8%) had biological prosthetic valve endocarditis and 3 (6.3%) mechanical prosthetic valve; 40 (45.5%) patients had the disease in their native valve. The mean EuroSCORE II was 8.9±6.5%, and the main surgical indication was refractory heart failure in 38 (43.2%) patients. A total of 68 bioprosthesis (36 aortic, 32 mitral) and 29 mechanical prostheses (12 aortic, 17 mitral) were implanted and three mitral valve plasties performed. A total of 25 (28.4%) patients underwent double or triple valve procedures. Aortic annulus reconstruction by abscess was performed in 18 (20.5%) and six (6.81%) patients had combined procedure. The mean surgery time was 359±97.6 minutes.

**Results:**

The overall survival in up to a 10-year follow-up period was 79.5%. In the univariate analysis, the main mortality predictors were positive blood cultures (*P*=0.003), presence of typical microorganisms (*P*=0.008), most frequently *Streptococcus viridans* (12 cases; 25%); C-reactive protein (hazard ratio [HR] 1.034, 95% confidence interval [CI] 1.000 to 1.070, *P*=0.04); creatinine clearance (HR 0.977, 95% CI 0.962 to 0.993, *P*=0.005); length of surgery: every five minutes multiplies the chance of death 1.005-fold (HR 1.005, 95% CI 1.001 to 1.009, *P*=0.0307); age (HR 1.060, 95% CI 1.026 to 1.096, *P*=0.001); and EuroSCORE II (HR 1.089, 95% CI 1.030 to 1.151, *P*=0.003).

**Conclusion:**

A positive blood culture with typical microorganism, C-reactive protein, age, EuroSCORE II, total surgical time and the presence of postoperative complications were the major predictors of mortality and significantly impacted survival in up to a 10-year follow-up period.

**Table:** 

Abbreviations, acronyms & symbols	
AF	= Atrial fibrillation
AFRVR	= Atrial fibrillation with rapid ventricular response
AIE	= Active infective endocarditis
BMI	= Body mass index
CI	= Confidence interval
CRP	= C-reactive protein
CKD	= Chronic kidney disease
DM	= Diabetes mellitus
HR	= Hazard ratio
IE	= Infective endocarditis
LVEF	= Left ventricular ejection fraction
PASP	= Pulmonary artery systolic pressure
PH	= Pulmonary hypertension
SH	= Systemic hypertension

## INTRODUCTION

Patients with valvulopathies, particularly those of rheumatic etiology, mitral valve prolapse with insufficiency, and degenerative aortic valve disease or those of bicuspid origin, cardiac valve prostheses, valvulopathies corrected with prosthetic materials, prior endocarditis episodes and congenital heart disease are considered at risk of developing severe infective endocarditis (IE)^[1]^.

The main indications for the surgical treatment of IE are refractory heart failure and annular abscess formation^[2,3]^. Other indications are persistent bacteremia after five to seven days of treatment with appropriate antibiotics, persistent vegetation or vegetation greater than 10 mm, severe valvular regurgitation, mycotic endocarditis and multiresistant microorganisms^[3,4]^.

The surgical treatment of IE involves valvular repair of lesions limited to leaflets, replacing the valve with a prosthetic or annular reconstruction with the use of a pericardial patch and Dacron prosthetic graft in cases of greater impairment, such as in the case of annular abscess^[5-7]^.

In terms of patients evolution with active infective endocarditis (AIE), surgery is a protective factor and has been associated with a significant reduction in mortality in the longterm follow-up of these patients when compared with patients who remained in clinical treatment^[8]^.

In patients with AIE in a native valve, congestive heart failure and emergency surgery are considered independent risk factors for mortality; emergency surgery is also an independent risk factor for patients with prosthetic endocarditis^[2]^.

The survival of patients undergoing surgical treatment of IE in the native valve is greater than that of those with prosthetic valve IE^[5,7,9]^. Independent postoperative mortality predictors also include paravalvular abscesses, *Staphylococcus aureus* as the etiologic agent and a left ventricular ejection fraction (LVEF) lower than 40%^[7]^.

Dohmen et al.^[10]^ reported a significant difference in hospital mortality in relation to gender, with 23% mortality in females *vs.* 15% in males (*P*=0.01). Furthermore, age, the involvement of several valves and the need for preoperative dialysis are also major mortality risk factors^[10]^.

In a study of patients with aortic valve IE, dialysis patients with chronic kidney disease (CKD) and those who needed aortic annulus reconstruction had high postoperative mortality^[6]^. Another study of patients with IE in an isolated mitral valve showed worse event-free survival in those who had prosthetic mitral valve endocarditis; furthermore, preoperative shock, *Staphylococcus aureus* infection and bioprosthesis implantation were independent predictors of all-cause mortality. These outcomes were associated with the fact that bioprosthetic patients were older than the group of patients with mechanical prostheses^[11]^.

The need for simultaneous aortic and mitral valve intervention is common in IE and is related to increased technical difficulty. Studies have reported high postoperative morbidity and mortality in the short and long terms in these patients, with hospital mortality rates of up to 15.6% and late mortality rates of 32.2%^[5]^.

However, in cases of endocarditis, early intervention, *i.e.*, within the first 48 hours of hospitalization, has resulted in significant reductions in embolic events and all-cause mortality when compared with a conventional treatment group^[12]^.

In-hospital mortality of AIE patients undergoing surgical treatment is high as a result of sepsis development^[3]^. Preoperative, intraoperative and postoperative factors are associated with worse survival. This study identifies mortality predictors in the surgical treatment of AIE in a long-term follow-up.

## METHODS

This prospective observational study involved 88 consecutive adult patients with a definite or possible diagnosis of IE according to the modified Duke criteria^[13]^ who underwent surgery between January 2005 and December 2015. The clinical follow-up was concluded in December 2016, with a loss of 9.09%. Patients under 18 years of age and those whose IE diagnosis was rejected by the modified Duke criteria were excluded. This study was approved by the Institutional Research Ethics Committee under number CAAE 62465816.2.0000.5462.

Data collection was conducted in regard to demographic characteristics, personal pathological history, predisposing heart disease, laboratory test results, blood cultures, echocardiography, risk stratification according to EuroSCORE II, analysis of the modified Duke criteria, classifying patients as having definitive or possible diagnoses, surgical intervention characteristics and early and late postoperative progress.

The primary outcomes were hospital mortality, late mortality and determination of the main factors associated with increased mortality. Secondary outcomes were reoperation rates due to recurrence of endocarditis in up to a 10-year follow-up period.

All patients underwent median sternotomy with extracorporeal circulation. Hypothermic cardiopulmonary bypass was established at 32ºC. All patients received central cannulation. The aorta was cross-clamped and cold blood or cold crystalloid (Custodiol^®^) cardioplegia was used for myocardial protection with antegrade flow. Transverse aortotomy was performed 3.5 cm above the right coronary ostium for aortic valve replacement. Radical debridement of infected tissue and aortic root abscess cavity was performed. Bovine pericardial or Dacron prosthetic graft was used for stabilization of the infected area and aortic annulus. Aortic annulus enlargement was performed using the Manouguian technique in small aortic annulus cases. The mitral valve was exposed through a right-sided left atriotomy, the basic surgical principle was radical resection of all infected valvular and subvalvular tissue, and mitral valve replacement or repair was performed when indicated. Right atriotomy was performed for the repair of the tricuspid valve (tricuspid valve annuloplasty). Associated procedure was performed in six patients, including epicardial permanent pacemaker implantation, atrioseptoplasty, ventriculoseptoplasty and subaortic membrane resection and radiofrequency ablation procedure.

### Statistical Analysis

Categorical variables are expressed as absolute and relative frequencies and continuous variables as means, medians and standard deviations, with minima and maxima when indicated. The Mann-Whitney test was used for comparisons between groups, the Kaplan-Meier and Cox Model methods were used for survival analysis and mortality predictors, and the Log-Rank Test was used to evaluate differences between the curves. A value of *P*<0.05 was considered statistically significant.

## RESULTS

Of the 88 patients, 58 (65.9%) were male, the mean age was 50.87±16.15 years (17-87 years) and the mean body mass index (BMI) was 24.86±3.72 kg/m^2^ (16.16 to 37.72 kg/m^2^, median 24.14 kg/m^2^). There was a history of rheumatic fever in 31 (35.2%) patients; prior endocarditis in four (4.5%) patients; prior cardiac surgery with prosthetic valve implantation in 48 (54.5%) patients, of whom 27 (56.30%) had the surgery at our institution and 21 (43.8%) at other centers; and 22 (45.80%) patients with intervals between prior surgery and new surgery of less than or equal to 1 year, and 26 (54.2%) patients with intervals of greater than one year. Bioprosthetic endocarditis was observed in 45 (93.8%) patients, and three (6.3%) patients had mechanical prosthetic endocarditis. The prosthesis of the majority of patients with prosthetic endocarditis was in the mitral position (23 patients; 47.9%), followed by the aortic position (20 patients; 41.7%), and there were only five (10.4%) patients with endocarditis in mitral and aortic prostheses simultaneously.

Native valve endocarditis occurred in 40 (45.5%) patients, most often in the mitral valve (19 patients; 47.5%); 13 (32.5%) cases occurred in the aortic valve and four (10%) in the aortic and mitral valves. Personal medical history findings were as follows: systemic hypertension (SH) was observed in 13 (14.8%) patients, diabetes mellitus (DM) in 17 (19.3%), and CKD in 17 (19.3%); six (6.8%) patients were on dialysis, 18 (20.5%) had atrial fibrillation (AF), four (4.5%) were intravenous drug users and seven (8%) had a permanent pacemaker. The mean EuroSCORE II was 8.9±6.5% (1.3 to 32.8%, median 6.8%).

Preoperative laboratory tests were as follows: mean hemoglobin, 11.19±2.26 mg/dL (6.6 to 16.8 mg/dL, median 11 mg/dL); leukocytes, 11.176±5.208 mm^3^ (2.300-32.500 mm^3^, median 10.375 mm^3^); mean C-reactive protein (CRP), 8.67±10.57 mg/dL (0.07 to 56 mg/dL, median 5.20); mean creatinine, 1.47±1.56 mg/dL (0.48 to 12.40 mg/L, median 1 mg/dL); mean creatinine clearance, 75.81±34.21 mL/min (7-168 mL/min, median 73 mL/min); and mean blood glucose, 108.03±40.34 mg/dl (61-331 mg/dl, median 97 mg/dL). Microbiological study revealed the following: 50 (56.8%) patients with positive blood cultures and typical microorganisms in 48 (54.5%) patients, most frequently *Streptococcus viridans* in 12 (25%) cases and *Staphylococcus epidermidis* in 10 (20.83%).

The main preoperative echocardiography findings were as follows: mean LVEF of 60.61±10.98% (24-84%, median 64%) and pulmonary hypertension (PH) in 62 (70.5%) patients, of which 23 (37.10%) cases were mild, 22 (35.50%) moderate and 17 (27.40%) severe. There was a mean pulmonary artery systolic pressure (PASP) of 48.09±17.56 mmHg (29-91 mmHg, median 46.50 mmHg). Vegetation was found in 66 (75%) patients, with a mean size of 13.77±6.7 mm (1.9 to 35 mm, median 12 mm); 50 (75%) patients had vegetation over 10 mm, and 15 patients had two or more vegetations. A total of 11 (12.5%) patients had abscesses.

The mean preoperative hospitalization time was 21±19.15 days (1-98 days, median 16 days). Emergency surgery (first 24 hours) was performed on five (5.7%) patients, urgent (24-48 hours) on 10 (11.4%) patients and elective (after 48 hours) on 73 (83%) patients. The principal surgical indication was refractory heart failure in 38 (43.2%) patients. There was a definitive diagnosis in 62 (70.5%) patients and a possible diagnosis in 26 (29.5%) according to the modified Duke criteria. The sample's main preoperative clinical characteristics are shown in Table 1.

**Table 1:** Preoperative clinical characteristics of the sample.

Variables		Total Population n=88	*P* value Mortality
Age, years		50.86±16.15	0.001
Gender	Male	58 (65.9%)	0.68
	Female	30 (34.1%)	
BMI, kg/m^2^		24.86±3.72	0.43
SH		13 (14.8%)	0.001
DM		17 (19.3%)	0.35
CKD		17 (19.3%)	0.11
Prior heart surgery		48 (54.5%)	0.48
Interval between prior and new surgery	≤ 1 year	22 (45.8%)	0.95
	> 1 year	26 (54.2%)	
IV drug users		4 (4.5%)	0.31
AF		18 (20.5%)	0.78
LVEF ≤50%		19 (21.59%)	0.21
PH≥50 mmHg		39 (44.3%)	0.32
NYHA	II	38 (43.2%)	0.84
	III	41 (46.6%)	
	IV	9 (10.2%)	
EuroSCORE II		8.9%±6.5%	0.03

AF=atrial fibrillation; BMI=body mass index; CKD=chronic kidney disease; DM=diabetes mellitus; IV=intravenous; LVEF=left ventricular ejection fraction; NYHA=New York Heart Association; PH=pulmonary hypertension; SH=systemic hypertension

A total of 68 (77.27%) bioprostheses (36 in the aortic position, 32 mitral), 29 (22.72%) mechanical prostheses (12 in the aortic position, 17 mitral) were implanted and three (3.4%) mitral valve plasties were performed. A total of 11 (12.5%) patients underwent procedures on the tricuspid valve, eight patients in combination with aortic or mitral surgery, and three (3.4%) had isolated tricuspid valve plasties. A total of 25 (28.4%) patients underwent double or triple valve procedures. Aortic annulus reconstruction by abscess with bovine pericardial patch or Dacron prosthetic graft was performed on 18 (20.5%) patients, aortic annulus enlargement was performed using the Manouguian technique in three (3.4%) patients and mitral valve annulus reconstruction was performed on four (4.5%). Only six (6.81%) patients had a combined procedure, epicardial permanent pacemaker implantation in three patients, atrioseptoplasty in one patient, ventriculoseptoplasty and subaortic membrane resection in one patient and surgical ablation of AF in one patient. The mean surgery time was 359±97.6 min (180-840, median 351 min)

**Fig. 1:**
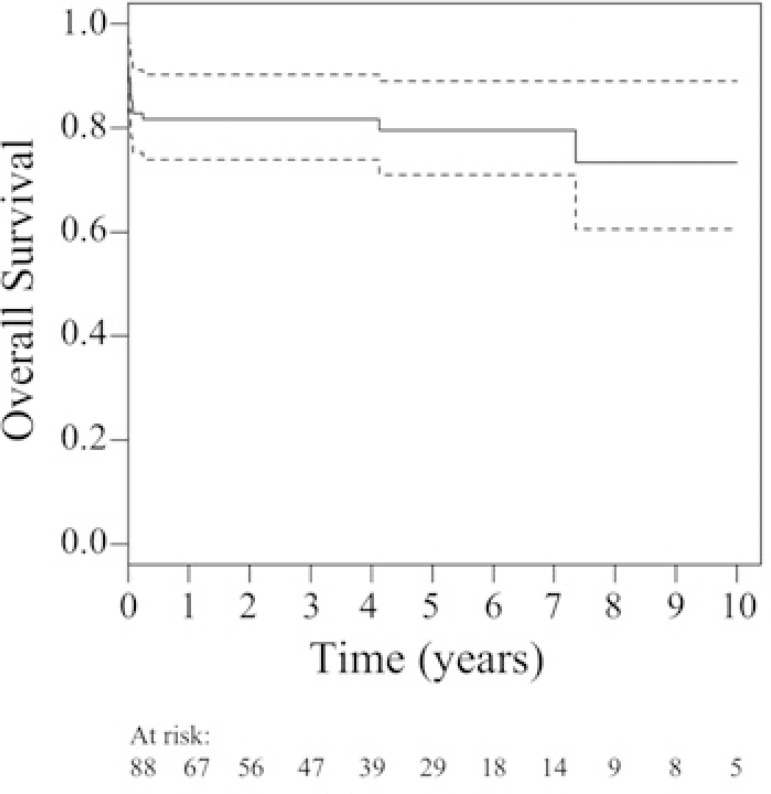
Survival curves of patients with active infective endocarditis undergoing surgical treatment.

The mean follow-up time was 4.5±3 years (0.04 to 11.6 years), and the mean survival time was 9.06±0.54 years (7.99 to 10.30 years). The overall survival in up to a 10-year follow-up period was 79.5%. There was in-hospital mortality within 30 days in 17.05% (15 patients); the leading cause of death was sepsis, in 13 (86.7%) patients (*P*=0.0001); late mortality was observed in 3.45% (3 patients) due to cardiogenic shock (*P*=0.0001) and one patient after reoperative complications regarding mitral and aortic bioprosthesis dysfunction 7.34 years after implantation (Figure 1).

The mean total hospitalization time was 47.25 days (interquartile range: 25.50 to 57.75 days) and was not significant in regard to mortality (*P*=0.48).

In the univariate analysis, the major mortality predictors in the preoperative period were positive blood cultures (32% *vs.* 6.25%, *P*=0.003); the presence of typical microorganisms (31.25% *vs.*. 7.5%, *P*=0.008); CRP, in which each 1 mg/dL increased the chance of death 1.034-fold (hazard ratio [HR] 1.034, 95% confidence interval [CI] 1.000 to 1.070, *P*=0.04); creatinine, in which each 1 mg/dL increased the chance of death 1.291-fold (HR 1.291, 95% CI 1.108 to 1.504, *P*=0.01); and creatinine clearance, in which each increase of 1 mL/min decreased the chance of death 0.977fold (HR 0.977, 95% CI 0.962 to 0.993, *P*=0.005). Other factors that demonstrated statistically significant relationships with mortality were SH (61.54% *vs.* 13.3%, *P*=0.0001); age, in which each year multiplied the chance of death 1.060-fold (HR 1.060, 95% CI 1.026 to 1.096, *P*=0.001), with a significant correlation between SH and age (*P*=0.0002); and EuroSCORE II, in which each 1% increased the chance of death 1.089-fold (HR 1.089, 95% CI 1.030 to 1.151, *P*=0.003).

In the postoperative period, 45 (51.1%) patients had complications, with a significant impact on mortality (37.78% *vs.* 2.32%, *P*=0.001); sepsis was observed in 19 (21.6%) patients (89.47% *vs.* 1.44%, *P*=0.001); shock in 17 (19.3%) patients (94.12% *vs.* 2.81%, *P*=0.001), with mixed shock (cardiogenic and septic) in eight (9.1%), septic shock in seven (8%) and vasoplegic shock in two (2.3%) patients; atrial fibrillation with rapid ventricular response (AFRVR) was observed in 15 (17%) patients (0 *vs.* 22.78%, *P*=0.133); CHB in five (5.7%) patients (40% *vs.* 19.27%, *P*=0.211); ventricular arrhythmia in four (4.5%) patients (25% *vs.* 20.23%, *P*=0.791); permanent pacemaker in three (3.4%) patients (33.33% *vs.* 20%, *P*=0.465); surgical site infection in three (3.4%) patients (0 *vs.* 15.30%, *P*=0.449); embolic events in two (2.27%) patients; septic pulmonary embolism in 1 patient (100% *vs.* 0, *P*=0.003); and ischemic stroke in one patient (0 *vs.* 15.66%, *P*=0.650). The anatomopathological results with signs of IE occurred in 35 (39.8%) patients (25.71% *vs.* 16.98%, *P*=0.222) (Table 2).

**Table 2:** Postoperative complications.

Variable	Population	Death n=18		*P* value
	total n=88	Presence of variable	Absence of variable	
Postoperative complication	45 (51.1%)	17 (37.78%)	1 (2.32%)	0.001
Sepsis	19 (21.6%)	17 (89.47%)	1 (1.44%)	0.001
Shock	17 (19.3%)	16 (94.12%)	2 (2.81%)	0.001
AFRVR	15 (17%)	__	18 (22.78%)	0.133
CHB	5 (5.7%)	2 (40%)	16 (19.27%)	0.211
Ventricular arrhythmia	4 (4.5%)	1 (25%)	17 (20.23%)	0.791
Permanent pacemaker	3 (3.4%)	1 (33.33%)	17 (20%)	0.465
Septic pulmonary embolism	1 (1.13%)	1 (100%)	__	0.003
Ischemic stroke	1 (1.13%)	__	18 (15.66%)	0.650
Surgical site infection	3 (3.4%)	__	18 (15.30%)	0.449

AFRVR=atrial fibrillation with rapid ventricular response; CHB=complete heart block

However, other variables evaluated in the univariate analysis showed no statistically significant differences in mortality, as follows: native valve endocarditis or that in a prosthetic valve (17.95% *vs.* 22.44%, *P*=0.55); interval between prior and new surgery ≤1 year or >1 year (22.72% *vs.* 23.07%, *P*=0.95); presence of abscesses (18.18% *vs.* 20.77%, *P*=0.98%); presence of vegetation (19.70% *vs.* 22.72%, *P*=0.76); emergency, urgent and elective surgery categories (20%, 10% and 21.92%, respectively, *P*=0.76); the need for annular reconstruction (27.78% *vs.* 18.57%, *P*=0.263); definitive or possible diagnosis according to the modified Duke criteria (24.19% *vs.* 24.44%, *P*=0.214); valve position: mitral *vs.* aortic *vs.* tricuspid *vs.* mitral with aortic and tricuspid *vs.* mitral with aortic *vs.* mitral with tricuspid (33.33% *vs.* 17.65% *vs.* 20% *vs.* 14.29% *vs.* 21.88% *vs.* 66.67%, *P*=0.55); number of implanted valves single *vs.* double *vs.* triple (20.63% *vs.* 18.18% *vs.* 33.33%, *P*=0.87) and isolated *versus* combined procedures (19.51% *vs.* 33.33%, *P*=0.42).

In the multivariate analysis, when the preoperative variables positive blood culture, presence of typical microorganisms, CRP, creatinine clearance, SH and age were compared, it was evident that SH was a risk factor (HR 5.194, 95% CI 1.268 to 21.266, *P*=0.022) and that creatinine clearance offered a greater mortality protection factor (HR 0.978, 95% CI 0.958 to 0.998, *P*=0.035) (Table 3).

**Table 3:** Multivariate analysis of preoperative factors.

Variable	HR	95% CI		*P *value
		Lower	Upper	
Positive blood culture	4.92	0.38	63.849	0.223
Typical microorganism	1.304	0.154	11.064	0.808
CRP	1.002	0.961	1.031	0.784
Creatinine clearance	0.978	0.958	0.998	0.035
SH	5.194	1.268	21.266	0.022
Age	0.997	0.951	1.046	0.915

CI=confidence interval; CRP=C-reactive protein; SH=systemic hypertension; HR=hazard ratio

The reoperation rate was 6.8% (6 patients) in up to a 10year follow-up period, with a mean time free of reoperation of 5.22±3.83 years (1.02-9.34 years, median 5.11 years); the causes of reoperation were recurrence of endocarditis in three (50%) patients, bioprosthetic valve dysfunction in two (33.33%) and new valve lesion in one (16.66%).

**Fig. 2:**
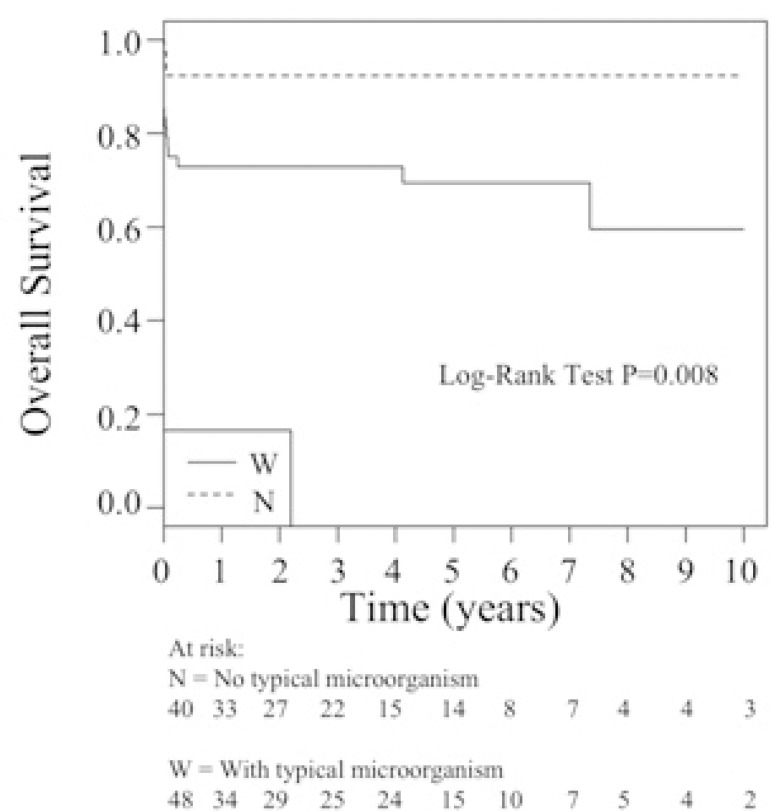
Survival curves of patients with active infective endocarditis undergoing surgical treatment with and without typical microorganisms in their preoperative blood cultures.

**Fig. 3:**
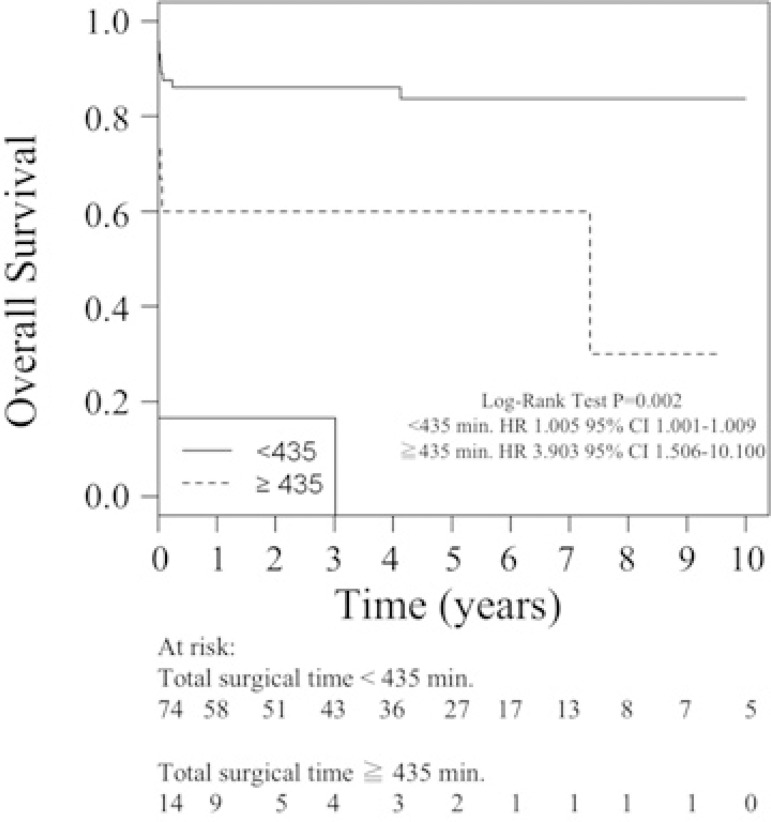
Survival curves of patients with active infective endocarditis undergoing surgical treatment with total surgery times less than 435 minutes and with total surgery times greater than or equal to 435 minutes.

Survival curves of patients with AIE undergoing surgical treatment with and without typical microorganisms in their preoperative blood cultures and with total surgery times less than 435 minutes and with total surgery times greater than or equal to 435 minutes are present in Figures 2 and 3, respectively.

## DISCUSSION

Surgical valve treatment for AIE is associated with high hospital mortality, which is a consequence of complications arising from IE, especially heart failure, persistent infection and recurrent embolism, which in turn have significant impacts on survival^[14]^.

In a study of 449 patients with left-sided IE, 240 patients underwent heart valve surgery. Surgical treatment was associated with high short-term mortality (in the first 14 days post-operatively) (HR 3.69, 95% CI 2.17 to 6.25, *P*<0.0001) and with decreased long-term mortality (HR 0.55, 95% CI 0.35 to 0.87, *P*=0.01)^[8]^. Despite the high hospital mortality, surgical treatment is considered a protective factor in long-term survival analysis when compared with clinical treatment.

In our series, a mortality rate within the first 30 postoperative days of 17.05% and an overall mortality of 20.50% were demonstrated. These results are compatible with those reported by Casabé et al.^[2]^ in a study of 186 IE patients, who found early mortality in 21.8% and an overall mortality of 22.6%. Machado et al.^[15]^ found a 30-day mortality of 17%, and Gatti et al.^[16]^ reported an in-hospital mortality of 20.3%.

Preoperative, intraoperative and postoperative factors are associated with a worse prognosis in terms of both short- and long-term survival^[17]^. Our study of the major predictors of preoperative mortality revealed the presence of positive blood cultures (*P*=0.003) and typical microorganisms (*P*=0.008). Among the latter, the most frequent were *Streptococcus viridans* in 12 (25%) cases and *Staphylococcus epidermidis* in 10 (20.83%); these findings are similar to those described in the literature^[2,18,19]^. In this same regard, *Staphylococcus aureus* has consistently been defined as an independent risk factor for hospital death (odds ratio [OR] 2.6, 95% CI 1.01 to 4.20)^[2,18,20,21]^.

A study with patients on chronic hemodialysis undergoing aortic valve replacement for AIE found a high mortality rate of 42.2% within the first 30 days postoperatively^[6]^. In a study by Park et al.^[17]^, dialysis was a predictor of mortality in 6 months. In our sample, only six (6.8%) patients had a history of CKD with hemodialysis, which showed no statistically significant association with mortality (*P*=0.11); however, when the creatinine and creatinine clearance variables were analyzed, statistically significant relationships were found (HR 1.291, 95% CI 1.108 to 1.504, *P*=0.01 and HR 0.977, 95% CI 0.962-0.993, *P*=0.005, respectively).

There has been a preponderance of males in the population of patients undergoing IE surgery^[2,7,15]^. In our study, a predominantly male population of 58 (65.9%) could be noted, consistent with the literature findings.

In this context, Dohmen et al.^[10]^ evaluated long-term surgical results based on gender in patients with AIE in the aortic valve and found a significant difference in early mortality rate (15% among men and 23% among women, *P*=0.01). This finding was not consistent with our results, which showed no significant difference according to gender (*P*=0.68).

In evaluating elapsed time before surgery, in a study comparing early (before 48 hours) and conventional surgical treatment (after 48 hours), Kang et al.^[12]^ observed no statistically significant difference between groups in terms of mortality from all causes (*P*=0.59). In another study with 132 patients, Kim et al.^[22]^ also demonstrated no statistically significant difference in the short- and long-term survival rates of up to 5 years of followup in early and conventional surgery subgroups. Similar results to those reported in our study compared emergency (within 24 hours), urgent (the first 48 hours) and elective (after 48 hours) surgeries and found no statistically significant differences in mortality (*P*=0.76).

However, Casabé et al.^[2]^ demonstrated that an urgent surgical indication was associated with a high mortality rate (*P*<0.001) in a prosthetic valve endocarditis subgroup with an interval of ≤1 year between the surgeries.

Age and postoperative complications have emerged as mortality predictors^[7,15,17,20,23]^. These findings are in agreement with our results; in terms of age, every extra year increased the chance of death 1.060-fold (HR 1.060, 95% CI 1.026 to 1.096, *P*=0.001), and the presence of postoperative complications was associated with a statistically significant difference in mortality rate (37.78% *vs.* 2.32%, *P*=0.001). In our experience, a history of SH had a significant impact on mortality, both in the univariate and multivariate analyses (*P*<0.05); however, a significant association between SH and age could be determined (*P*=0.0002).

In a study by Cresti et al.^[20]^, septic shock and persistent bacteremia were independent mortality predictors, consistent with our results, where shock significantly increased in-hospital mortality rates (94.12% *vs.* 2.81% *P*=0.001). Preoperative hematological parameters are related to poorer prognosis in IE. In a study of 62 consecutive patients with IE procalcitonin (*P*=0.030) and platelet/lymphocyte ratio (*P*=0.008) all significantly affected mortality^[24]^, as in our study: in terms of CRP, each 1 mg/ dL increased the chance of death 1.034-fold (HR 1.034, 95% CI 1.000 to 1.070, *P*=0.04).

In current clinical practice, the EuroSCORE II is considered a useful tool in perioperative risk assessment of AIE patients and offers good predictive performance^[25]^. In our experience, EuroSCORE II (HR 1.089, 95% CI 1.030 to 1.15, *P*=0.003) was a significant predictor of mortality.

Intraoperative factors are also related to lower survival. In this regard, one study found associations between both extracorporeal circulation time (*P*<0.0001) and aortic clamping time (*P*=0.0005)^[16]^; these findings agree with our experience, where length of surgery had a significant impact: every 5 minutes increased the chance of death 1.005-fold (HR 1.005, 95% CI 1.001 to 1.009, *P*=0.0307), and from 435 minutes, every 5 minutes increased the chance of death 3.903-fold (HR 3.903, 95% CI 1.508 to 10.100, *P*=0.005).

Our study was limited by evaluating patients treated at a single center, which specializes in the treatment of cardiovascular disease and is considered a referral center; it serves a larger number of patients with cardiovascular comorbidities, among whom there is a high frequency of prior heart surgery. This frequency has an impact on surgical risk, which explains the high hospital mortality rate. However, our results are consistent with those reported by other studies. A total of 48.3% of patients with prior cardiac surgery came from other centers, making it difficult to determine the actual times between presentation of the clinical condition, diagnosis and surgical treatment.

Because of the observational nature of the study design, the evaluation of laboratory parameters performed routinely in the institution in the care of endocarditis patients was limited, and it should be noted that there are other hematologic parameters that predict risk.

## CONCLUSION

The main mortality predictors in the univariate analysis were, preoperatively, positive blood culture, typical microorganism, CRP, creatinine clearance, age, SH and EuroSCORE II; in the intraoperative period, total surgery time; and postoperatively, the presence of complications, shock, sepsis and pulmonary embolism. These factors significantly impacted short-term survival in up to a 10-year follow-up period in patients undergoing surgical treatment of AIE.

In the multivariate analysis, when the preoperative predictors positive blood culture, typical microorganism, age, SH, creatinine clearance and CRP were compared, only SH was a risk factor for mortality, creatinine clearance was a protective factor and SH showed a significant association with age.

**Table:** 

Authors' roles & responsibilities	
JLRO	Substantial contributions to the conception or design of the work; or the acquisition, analysis, or interpretation of data for the work; drafting the work or revising it critically for important intellectual content; final approval of the version to be published
MAS	Substantial contributions to the conception or design of the work; or the acquisition, analysis, or interpretation of data for the work; drafting the work or revising it critically for important intellectual content; final approval of the version to be published
RTA	Drafting the work or revising it critically for important intellectual content; final approval of the version to be published
AR	Drafting the work or revising it critically for important intellectual content; final approval of the version to be published
DDT	Drafting the work or revising it critically for important intellectual content; final approval of the version to be published
SKG	Drafting the work or revising it critically for important intellectual content; final approval of the version to be published
RTMK	Drafting the work or revising it critically for important intellectual content; final approval of the version to be published
LCBS	Agreement to be accountable for all aspects of the work in ensuring that questions related to the accuracy or integrity of any part of the work are appropriately investigated and resolved; final approval of the version to be published
